# RSA-TransUNet: a robust structure-adaptive TransUNet for enhanced road crack segmentation

**DOI:** 10.3389/fnbot.2025.1633697

**Published:** 2025-09-16

**Authors:** Liling Hou, Fei Yu, Yaowen Hu, Yang Hu, Ruoli Yang

**Affiliations:** 1 Liling Hou Zhangzhou Institute of Technology, Zhangzhou, China; 2 College of Physics and Information Engineering, Minnan Normal University, Zhangzhou, China; 3 Key Lab of Light Field Manipulation and System Integration Applications in Fujian Province, School of Physics and Information Engineering, Minnan Normal University, Zhangzhou, China; 4 Key Lab of Intelligent Optimization and Information Processing, Minnan Normal University, Zhangzhou, China; 5 College of Computer Science and Technology, National University of Defense Technology, Changsha, China; 6 College of Electronic Information and Physics, Central South University of Forestry and Technology, Changsha, China; 7 College of Systems Engineering, National University of Defense Technology, Changsha, China

**Keywords:** road crack segmentation, axial-shift MLP attention, adaptive spline linear unit, structure-aware multi-stage, evolutionary optimization

## Abstract

With the advancement of deep learning, road crack segmentation has become increasingly crucial for intelligent transportation safety. Despite notable progress, existing methods still face challenges in capturing fine-grained textures in small crack regions, handling blurred edges and significant width variations, and performing multi-class segmentation. Moreover, the high computational cost of training such models hinders their practical deployment. To tackle these limitations, we propose RSA-TransUNet, a novel model for road crack segmentation. At its core is the Axial-shift MLP Attention (ASMA) mechanism, which integrates axial perception with sparse contextual modeling. Through multi-path axial perturbations and an attention-guided structure, ASMA effectively captures long-range dependencies within row-column patterns, enabling detailed modeling of multi-scale crack features. To improve the model’s adaptability to structural irregularities, we introduce the Adaptive Spline Linear Unit (ASLU), which enhances the model’s capacity to represent nonlinear transformations. ASLU improves responsiveness to microstructural variations, morphological distortions, and local discontinuities, thereby boosting robustness across different domains. We further develop a Structure-aware Multi-stage Evolutionary Optimization (SMEO) strategy, which guides the training process through three phases: structural perception exploration, feature stability enhancement, and global perturbation. This strategy combines breadth sampling, convergence compression, and local escape mechanisms to improve convergence speed, global search efficiency, and generalization performance. Extensive evaluations on the Crack500, CFD, and DeepCrack datasets—including ablation studies and comparative experiments—demonstrate that RSA-TransUNet achieves superior segmentation accuracy and robustness in complex road environments, highlighting its potential for real-world applications.

## 1 Introduction

Road construction is one of the most critical infrastructure projects globally, playing a key role in public welfare. Road safety is a fundamental factor in ensuring safe driving and smooth traffic conditions (Sohail et al., 2023). If cracks on road surfaces are not repaired promptly, they may enlarge over time, and under the influence of natural elements such as rainfall, erosion may penetrate the roadbed, potentially leading to localized road collapses, thereby posing significant traffic safety hazards. Studies indicate that traffic accidents caused by road cracks are common occurrences each year, highlighting the importance of timely and efficient crack repair (Deme, 2020). Cracks, as a typical pavement hazard, pose a significant threat to road integrity, and repairing these cracks is essential to maintaining optimal road conditions (Zhang et al., 2024). Currently, crack image segmentation is a vital research direction within the field of computer vision, providing a fundamental basis for the understanding and analysis of road crack images. Moreover, it offers crucial support for various crack image processing tasks and applications (Kyem et al., 2025). During the crack repair process, accurate crack segmentation is required to assess the boundaries of crack regions, thereby enabling the extraction of precise crack information, which is essential for conducting scientifically informed repair efforts (Khan et al., 2025).

Early researchers primarily employed machine learning approaches to conduct crack segmentation studies. However, with the continuous expansion of crack datasets, the research on cracks has become increasingly complex, diverse, and constrained (Liu et al., 2025). To advance crack segmentation technology, extensive exploration has been undertaken by researchers. Deng et al. (2023) proposed an improved residual unified network algorithm for precise pixel-level segmentation within crack regions, effectively addressing the challenges related to the pixel representation of crack width and length. Chen et al. (2022) proposed a method for detecting potential crack regions in pavement using adaptive thresholding. The algorithm combines both global and local thresholds to segment the image, effectively reducing image noise. Hang et al. (2023) introduced an attention-based feature fusion network, which utilizes efficient channel attention and feature fusion at each decoder layer to assist in the recovery of low-level features for crack localization. This approach achieved a detection accuracy of 84.49% for automatic pixel-level detection of concrete cracks. Tao et al. (2023) developed a novel convolutional transformer network based on an encoder-decoder architecture to address the challenges posed by the elongated and sharp topological features of crack as, as well as complex backgrounds. This model enables improved segmentation by focusing on the local details of cracks and adjusting the feature sizes of other components as needed. Tabernik et al. (2023) proposed a novel deep learning model that integrates pixel-level segmentation with image-level classification, effectively addressing the challenges of capturing both local and broader contextual information. This model enables automated quality control of concrete surfaces during the construction and maintenance stages. Chu and Chun (2024) introduced a multi-scale crack feature extraction network that employs two cascading operations to achieve collaborative enhancement. By incorporating strip pooling operations, the network improves the representation of both transverse and longitudinal crack pixels in complex backgrounds.

In addition to the aforementioned methods for enhancing crack features, several researchers have also addressed the issue of boundary segmentation ambiguity. Li et al. (2022) proposed a dense boundary refinement network that combines the advantages of short-term dense cascading networks and refinement networks. By eliminating redundant structures, the model improves detection rates and optimizes crack details using binary cross-entropy and Dice loss functions. Chen et al. (2023) modeled cracks as sub-cracks with corresponding orientations and introduced a directional sub-crack detector. This approach employs multi-branch angular regression loss to simultaneously learn the direction and variance of sub-cracks, thereby addressing the discontinuity of crack boundaries and the ambiguity of sub-crack orientations. Liu et al. (2022) proposed a bridge crack segmentation method based on a densely connected U-Net network, which incorporates a background elimination module and a cross-attention mechanism. This approach effectively enhances the primary features of small cracks and utilizes varying weights to retain redundant information. Lin et al. (2023) introduced a deep multi-scale crack feature learning model for crack segmentation. By employing hybrid dilated convolutions to increase the receptive field, the model captures more crack information. The included multilayer perceptron then transforms high-dimensional crack features into lower-dimensional representations, reducing parameters and enhancing the network’s robustness to noise.

It is noteworthy that existing lightweight methods often face challenges such as low computational efficiency, complex crack patterns, and difficult backgrounds, which result in inaccurate detection and make them impractical for real-world applications. To address these limitations, Zim et al. (2025) proposed a crack segmentation model that deeply integrates depthwise separable convolutional layers and mobile vision modules to capture both global and local features, enabling precise crack segmentation. Zhang et al. (2023) introduced an efficient crack segmentation neural network that incorporates small-kernel convolutional layers, along with parallel max pooling and convolution operations, into the architecture for fast crack feature extraction and model parameter reduction, thereby accelerating real-time pavement crack detection and segmentation without compromising performance. Despite significant advancements in the field of crack segmentation, the diversity of cracks under various environmental conditions presents a considerable challenge, making it difficult for existing single models to overcome issues such as unreliability, lack of robustness, and low trustworthiness. These challenges remain substantial barriers to the practical application of crack segmentation models. In their research on crack segmentation, Ha et al. (2022) incorporated five types of crack features (alligator crack, longitudinal crack, transverse crack, pothole, and patching) to address the requirements of real-world road scenarios. By employing separately trained linear CrackU-Nets, they achieved a segmentation accuracy of 91.2% on input images. In summary, while the aforementioned methods have investigated issues related to segmentation accuracy and model training costs, several limitations remain: (1) the models exhibit a lack of sensitivity to texture variations during crack segmentation, leading to blurred edges and suboptimal segmentation accuracy; (2) existing models demonstrate high accuracy for specific datasets but suffer from reduced robustness when dealing with a diverse range of crack types; (3) the growing volume of large-scale crack data has resulted in high training costs, which remain inadequately addressed. To tackle these challenges, we have conducted extensive research, and the main contributions of this paper are as follows:

1. We propose the Axial-Shift Multi-Path Attention (ASMA) mechanism, which enables fine-grained modeling of multi-scale crack texture features. This approach addresses the challenges of edge blurring, weak textures, and significant width variations in crack images, which contribute to the difficulty of segmentation. The ASMA module integrates axial shift perception with sparse contextual modeling to enhance attention. By introducing directional perception through multi-path axial perturbation operations and embedding a Criss-Cross spatial attention mechanism within each path, the module captures long-range dependencies in both row and column structures. Subsequently, a channel dynamic fusion mechanism adaptively integrates the three feature paths. ASMA effectively enhances the model’s ability to capture fine-grained structural details and multi-scale textures of crack regions, while maintaining a lightweight architecture. It is particularly well-suited for the representation of irregularly shaped crack structures.
2. We propose the Adaptive Spline Linear Unit (ASLU), a novel linear transformation module designed to enhance structural representation. This module addresses the limitations of traditional linear layers in modeling complex structures and distribution shifts in high-heterogeneity crack images. The ASLU, which can be seamlessly integrated into various neural network architectures, consists of two components: a basic linear path that preserves computational efficiency and linear combination capabilities, and a spline kernel path that constructs a local kernel response mechanism based on B-spline basis functions. This enables effective modeling of nonlinear structural variations across input dimensions. Through the additive fusion of two distinct paths, the ASLU achieves a high-response representation of microstructural variations, morphological distortions, and local discontinuities, while maintaining a compact architecture. This significantly enhances the model’s ability to fit complex crack structures and improves its robustness across diverse domains.
3. To enhance the robustness and ability to escape local optima during model training, we propose a Structure-aware Multi-stage Evolutionary Optimization (SMEO) strategy. This approach addresses common challenges such as premature convergence to local optima, insufficient feature representation, and difficulties in achieving convergence across diverse data domains. SMEO consists of a three-stage evolutionary optimization strategy that simulates structural state transitions. The method is divided into three phases: structure-aware exploration, feature stabilization and fine-tuning, and global perturbation escape. These phases correspond to broad sampling during the initial training stage, convergence compression in the mid-stage, and local escape in the later stage. Each phase employs differentiated position update formulas and perturbation mechanisms, dynamically switching behavior modes based on feature response states, thereby enhancing the model’s global search capability, local stability, and generalization adaptability during training.
4. We conducted extensive experiments to validate the effectiveness and advanced performance of the network. First, an ablation study was performed on RSA-TransUNet to assess the contribution of each module. Next, the model was compared with seven state-of-the-art crack segmentation networks to demonstrate its superiority. Furthermore, to evaluate the generalization ability of each model, generalization experiments were carried out on three public datasets. The experimental results demonstrate that the proposed method exhibits high stability and can accurately segment fine textures and multi-class road cracks in complex scenarios. RSA-TransUNet offers an effective tool for road crack detection and provides a better solution for mitigating traffic safety hazards.

## 2 Materials and methods

### 2.1 Materials

This study utilized the following public datasets: Crack500 (Yang et al., 2019), DeepCrack (Zou et al., 2018), and CFD (Shi et al., 2016), which are described in detail below.

#### 2.1.1 Crack500

The Crack500 dataset consists of 500 images, each with a resolution of 2,560 × 2,592 pixels. These images feature various crack shapes and widths against complex background textures, which include interference from shadows, lighting variations, and pavement stains. This complexity challenges the generalization ability of crack detection algorithms.

#### 2.1.2 DeepCrack

DeepCrack is a widely recognized dataset for evaluating crack detection algorithms. It consists of 537 images, each with a resolution of 384 × 544 pixels. The dataset features significant intensity differences between cracks and the background, including various types of cracks (e.g., longitudinal cracks, transverse cracks) under different environmental conditions (e.g., sunny, rainy). The background is also relatively complex, with noise interference, which aids in the effective identification of cracks in pavement images.

#### 2.1.3 CFD

The CFD Road Crack Dataset comprises 118 images that reflect the pavement conditions of urban roads in Beijing, China. Each image is manually annotated with the true ground contours. All images were captured using an iPhone 5, with a focal length of 4 mm, an aperture of f/2.4, and an exposure time of 1/134 s. The image width ranges from 1 to 3 mm. Notably, these images contain various types of noise, such as shadows, oil stains, and water marks.

**Figure 1:**
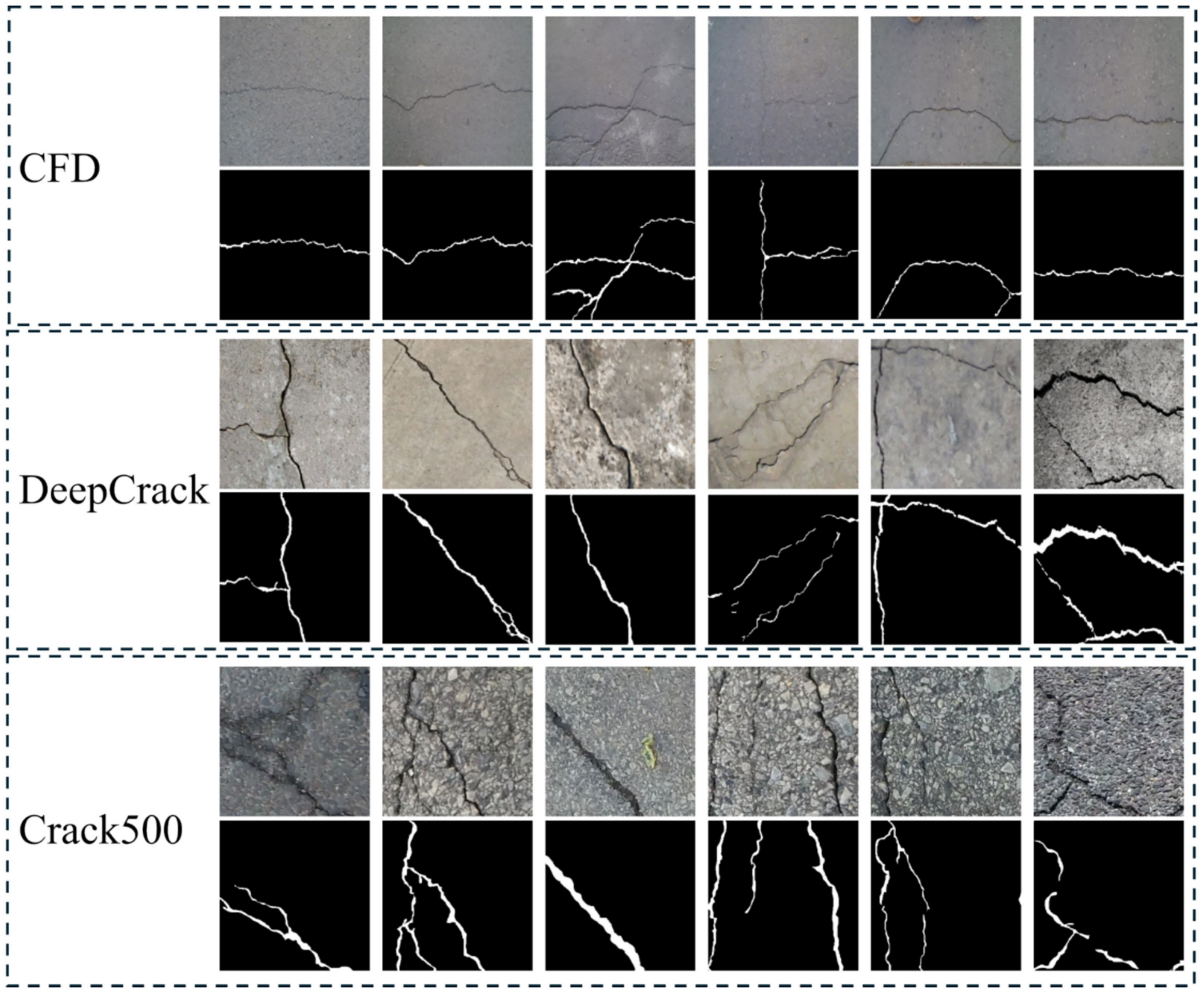
Sample images from the crack public dataset.

To ensure consistency and reproducibility of the experimental setup, we adopted a unified partitioning strategy for all three datasets: 70% as the training set, 10% as the validation set, and 20% as the test set. This partitioning method can ensure sufficient training while reasonably evaluating the model’s generalization ability on unseen samples (Figure 1).

### 2.2 Methods

In practical applications, crack images often present complex challenges, such as weak texture details, blurred boundaries, and significant background interference, making accurate pixel-level segmentation tasks particularly demanding. First, within fine-grained crack regions, crack edges frequently appear discontinuous, blurred, or nearly indistinguishable from the background, especially under environmental influences like dust contamination, surface weathering, and lighting shadows. These conditions lead to highly irregular variations in crack width. Additionally, in crack segmentation tasks, models are required to handle images of cracks from diverse materials, such as concrete and asphalt, captured using various devices, including smartphones and drones, in different construction environments like underground spaces or tunnels (Jin et al., 2025). This diversity introduces significant differences in data distribution, leading to a sharp decline in accuracy for many existing methods when applied to new scenarios, thereby hindering their generalization capability and severely affecting the feasibility of real-world engineering deployments. Furthermore, deep learning models exhibit a growing dependence on large and diverse datasets, resulting in increased computational resource consumption and longer training times. These factors limit the widespread application of such models in resource-constrained platforms or small sample scenarios.

In this study, we adopt the TransUNet framework, which integrates Transformer (Han et al., 2021) and U-Net (Ronneberger et al., 2015) architectures, as the backbone for achieving high-precision semantic segmentation of crack images. TransUNet combines the strengths of Convolutional Neural Networks (CNNs) in local feature extraction with the Transformer architecture’s capability to model long-range dependencies, demonstrating excellent segmentation accuracy in tasks such as medical image analysis and structural texture modeling. However, the original TransUNet architecture still faces challenges when applied to crack images, which possess fine-grained textures, blurred edges, and significant multi-scale variations. These challenges include insufficient structural representational power, weak cross-domain robustness, and low optimization efficiency.

**Figure 2:**
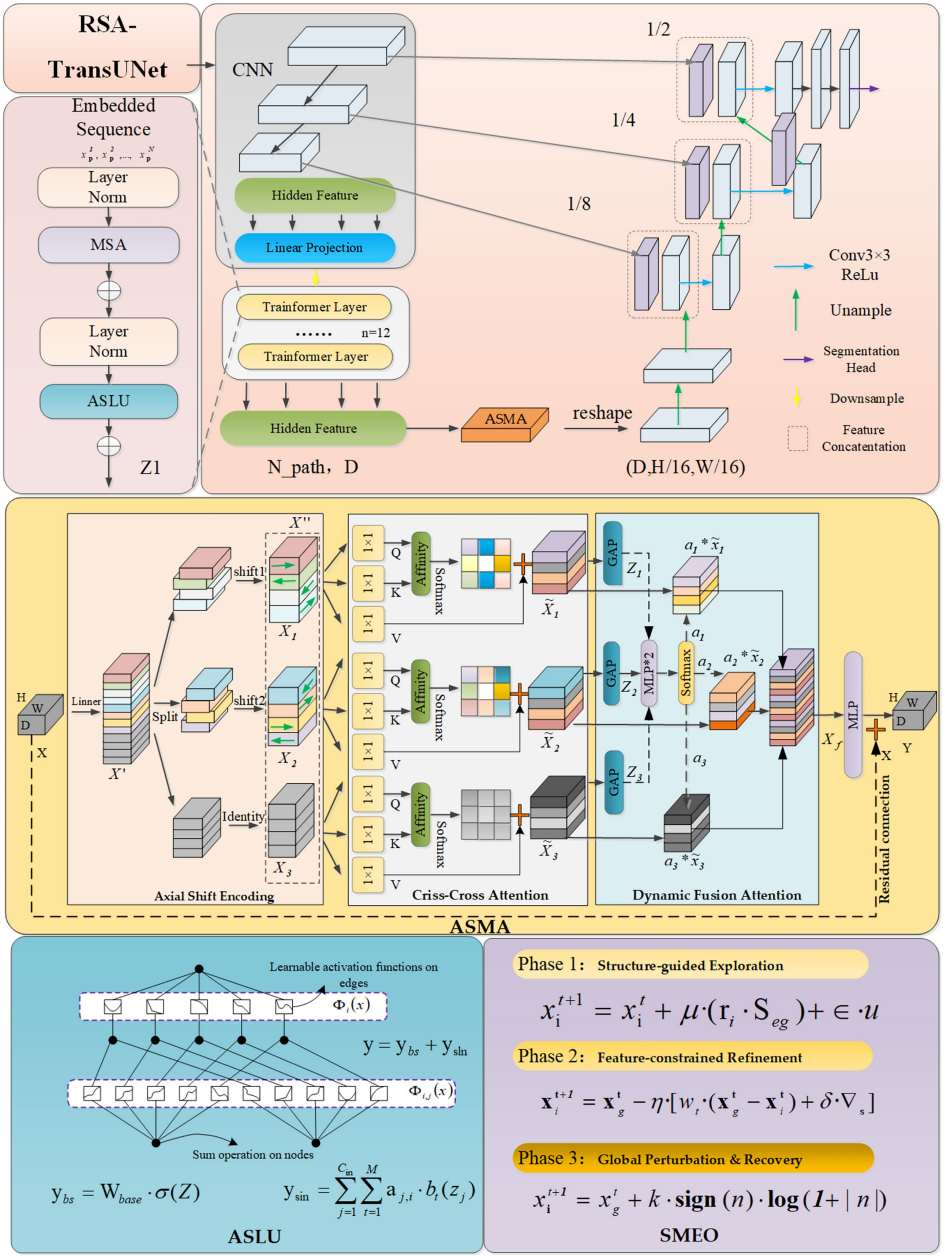
Block diagram of RSA-TransUNet structure.

To address the aforementioned issues, this paper proposes the RSA-TransUNet, where “RSA” stands for Robust Structure-Adaptive, reflecting the model’s ability to generalize across complex crack patterns and variable imaging conditions. Building upon the TransUNet backbone, we introduce three complementary structural improvements and optimization mechanisms, targeting attention enhancement, feature transformation expression enhancement, and training process optimization. These enhancements collectively form a unified framework for crack segmentation. The overall structure of the RSA-TransUNet crack image segmentation network is depicted in Figure 2.

Firstly, we introduce the ASMA module after the Transformer encoding block in the intermediate layer of TransUNet to enhance the global attention’s ability to capture local spatial texture structures. The ASMA module adopts a multi-path axial modeling architecture. The input features are initially divided into three sub-paths, each subjected to spatial translation along different directions to enhance directional perception. Subsequently, sparse horizontal and vertical spatial attention mechanisms are incorporated within each path, and long-range contextual dependencies are modeled through the Criss-Cross path, thereby strengthening the model’s understanding of structural relationships. The enhanced features from the three paths are then dynamically weighted and fused using a channel attention mechanism, adaptively integrating information across different directions and scales. Finally, a linear mapping and residual connection are employed to output the overall enhanced feature representation. Despite its lightweight structure, ASMA effectively unifies local detail perception and global semantic aggregation, significantly improving the segmentation accuracy and robustness of the model for crack-like objects.

Furthermore, to enhance the adaptability of the linear layers in TransUNet when confronted with structural deformations and variations in data distribution, we replace all standard linear projection layers in the original network—such as Patch Embedding, MLP layers, and the fully connected layers in the Decoder—with the newly developed ASLU (Adaptive Spline Linear Unit). This module builds upon traditional linear transformation structures and incorporates a nonlinear local response pathway based on B-spline kernel functions, enabling the model to adapt to structural variations in the input. By combining the linear and kernel pathways additively, ASLU improves the model’s ability to capture complex structural responses, such as fractures, widening, and boundary blurring in different crack regions. This enhancement ultimately boosts the overall segmentation network’s expressiveness and stability.

Finally, during the model training phase, we replace the default optimizer AdamW in the backbone network with a custom-designed Structure-aware Multi-Stage Evolution Optimization strategy (SMEO) to enhance the model’s convergence and robustness across different data sources and training conditions. SMEO simulates the structural evolution process by incorporating three key stages: structure-aware exploration, feature-constrained fine-tuning, and perturbation-based jump recovery. These stages dynamically update the model parameters, effectively mitigating issues such as local optima, slow convergence, and accuracy fluctuations during training. Furthermore, SMEO enhances the model’s robust training performance in cross-domain scenarios.

In summary, this study enhances the TransUNet framework by introducing three modules—ASMA, ASLU, and SMEO—across the key stages of perceptual modeling, structural representation, and optimization training. These synergistic improvements significantly enhance the model’s structural adaptability and segmentation performance in complex crack imaging scenarios.

#### 2.2.1 Axial-shift MLP attention(ASMA)

In the field of road crack segmentation, we observe that the texture changes in the edge areas of small cracks are very small, especially when facing challenges such as blurry crack edges and blurry crack features. In this situation, human recognition is neither accurate nor efficient. Effectively addressing these issues can significantly enhance the saliency of crack feature maps, thereby improving the segmentation model’s ability to perceive fine details. Given that the essence of the above issues stems from the complexity of cracks in spatial direction and context dependence, we have developed a unified strategy that combines directional modeling and context awareness - the ASMA module. This strategy aims to enhance the feature expression ability from multiple scales and dimensions, and improve the perception accuracy of the model for complex crack structures. The main goal of the ASMA module is to enhance the modeling ability of the model for complex edges, subtle textures, and irregular line structures in crack images. The ASMA module adopts a processing strategy of “multi path axial modeling spatial context enhancement channel adaptive fusion,” which improves fine-grained structure perception while maintaining a balance between expressive power and computational efficiency. This method is particularly effective for high-resolution image scenes. The input feature map is represented as $X\in R^{H\times W\times C}$ . The processing flow of ASMA can be divided into the following four stages:

(1) Axial shift encoding

To enhance direction perception ability, we introduce two modeling paths with differentiated axial perturbation strategies in the ASMA module. To introduce directional perception capability, we first perform a linear transformation on the input feature map X, increasing the number of channels by three to obtain the feature map $X\prime \in R^{H\times W\times 3C}$ . For relevant details, see (Equations 1–11):


$$X\prime =\text{Linear}(X),X\prime \in R^{H\times W\times 3c}$$ (1)

Then we evenly divide X′ prime into three sub paths along the channel dimension: $X_{1}$ , $X_{2}$ , and $X_{3}$ , each with a size of $R^{H\times W\times 3C}$ . Then, spatial perturbation operations are introduced for different paths to capture structural features in different directions:


$$X\prime \prime =[X_{1},X_{2},X_{3}],X_{i}\in R^{H\times W\times C},i=1,2,3$$ (2)

- Path $X_{1}$ : Apply symmetrical axial perturbations to the divided channel groups. Specifically, the channel is divided into multiple groups and offset in equal but opposite directions along the horizontal positive direction (plane W axis) and horizontal negative direction (plane H axis) (as indicated by the green arrows in the figure: the first group rotates clockwise and the second group rotates counterclockwise alternately), forming a staggered symmetrical offset structure;
- Path $\;X_{2}$ : Apply symmetrical axial perturbations to the divided channel groups as well. However, each subgroup rotates in the opposite direction to $X_{1}$ (as indicated by the green arrow in the figure: the first group rotates counterclockwise and the second group rotates clockwise alternately), forming an opposite staggered symmetric offset structure;
- Path $X_{3}$ : Keep unchanged to preserve global structure and texture consistency.

There are differences in the perturbation direction between $\;X_{1}$ and $X_{2}$ , forming complementary structures that ultimately provide diverse and direction sensitive feature foundations for subsequent attention modeling along with the path $X_{3}$ that preserves the original information. The above-mentioned rotation disturbance method is different from traditional axial translation operations, as it is more like performing directional cyclic displacement in the spatial feature plane. The paths $X_{1}$ and $X_{2}\;$ are arranged in a staggered manner in the initial offset direction, forming a mirrored and staggered perturbation layout in the spatial structure, further enhancing the modeling ability of the model for crack morphology, direction changes, and texture mutation areas. The joint modeling of three paths provides high-quality feature representations with directional differences and expression redundancy for subsequent attention mechanisms.

(1) Criss-cross attention for each branch

After obtaining the three axial perception paths, ASMA further introduces a spatial context modeling mechanism on each path to enhance its ability to model long-range structural dependencies. For each path feature map $X_{i}$ , three sets of convolutions are first applied to generate the corresponding Query, Key, and Value representations:


$$Q_{i}=W_{i}^{Q}X_{i},K_{i}=W_{i}^{K}X_{i},V_{i}=W_{i}^{V}X_{i}$$ (3)

$$Q_{i},K_{i},V_{i}\in R^{H\times W\times d}$$ (4)

For any given position u=(h,w) in the feature map, attention pathways are established only along the corresponding row (the *h*-th row) and column (the w-th column), thereby forming a sparse Criss-Cross path set $Q_{i},K_{i},V_{i}\in R^{H\times W\times d}$


$$N_{u}=\{(h,j),\forall j\neq w\}\cup \{(i,w),\forall i\neq h\}$$ (5)

Within this path range, the similarity between the Query and Key is computed to obtain the attention weights:


$$a_{u,v}=\frac{\exp(Q_{u}\cdot K_{v}^{T})}{\sum_{v\in N_{u}}\exp(Q_{u}\cdot K_{v}^{T})}$$ (6)

Finally, the attention weights are used to perform a weighted sum of the Value, aggregating contextual information while preserving the feature response of the current position itself:


$$\tilde{X_{i}}(u)=\sum_{v\in N_{u}}a_{u,v}\cdot V_{i}(v)+X_{i}(u)$$ (7)

Each path of ${\tilde{X}}_{i}(\mu )$ aggregates multi-directional information, encompassing both directional dependency information and context-enhanced feature representations. The output is denoted as $\tilde{X_{1}},\tilde{X_{2}},\tilde{X_{3}}\in R^{H\times W\times C}$

(2) Path fusion and channel weighting attention

To further integrate the aforementioned three structurally enhanced features, ASMA introduces a channel-level dynamic fusion module. This module learns the relative importance of the three features based on channel descriptors and performs the integration in a weighted manner.

Specifically, global average pooling is first applied to each enhanced feature ${\tilde{X}}_{i}$ to extract the channel descriptor vectors:


$$z_{i}=\mathrm{GAP}(\tilde{X_{i}})\in R^{C},i=1,2,3$$ (8)

The three channel descriptors are concatenated to form a fused vector $z=[z_{1};;z_{2};;z_{3}]\in R^{3C}$ , which is then passed through two layers of shared MLP (Multilayer Perceptron) to generate three sets of channel-level fusion weights:


$$[a_{1},a_{2},a_{3}]=\text{Softmax}(\mathrm{MLP}(z))\in R^{3\times C}$$ (9)

The Softmax operation is applied along the path dimension for normalization, ensuring that the weights are competitive. Ultimately, the three features are aggregated through a weighted sum along the channel dimension to produce the fused feature map:


$$X_{f}=a_{1}\cdot \tilde{X_{1}}+a_{2}\cdot \tilde{X_{2}}+a_{3}\cdot \tilde{X_{3}}$$ (10)

$X_{f}$ effectively leverages semantic information from multiple directional paths while enhancing its adaptability to texture discontinuities and directional structures.

(3) Output mapping and residual connection

The fused feature map $X_{f}\in R^{H\times W\times C}$ undergoes a linear transformation to restore its expressive capacity, and is then subjected to a residual connection with the original input feature X, yielding the final output:


$$Y=X+\mathrm{MLP}(X_{f})$$ (11)

This ensures stable information propagation while enhancing gradient flow and expression consistency during the training process.

In summary, ASMA effectively integrates spatial axial modeling with context-aware perception within its overall structure, explicitly capturing the directional patterns of cracks and fine-grained texture details in images. By incorporating a multi-path axial displacement mechanism, sparse attention modeling within paths, and channel-level dynamic fusion strategies, ASMA achieves a unified approach to local structure modeling and global dependency aggregation. This significantly improves the model’s ability to handle complex crack features, such as edge blurring, texture discontinuity, and scale inconsistency. It provides a more efficient and reliable feature representation for the precise extraction of crack-like targets.

#### 2.2.2 Adaptive spline linear unit (ASLU)

In the cross-domain segmentation task of crack images, the model often faces challenges such as unstable input feature distributions and complex structural responses due to significant differences in data acquisition conditions, material textures, and lighting environments. Traditional linear mapping layers, such as fully connected layers, rely solely on fixed weight matrices, making it difficult to effectively model the complex nonlinear relationships between input dimensions. This limitation restricts the model’s expressive power and generalization performance under heterogeneous structures. To address this issue, we propose the Adaptive Spline Linear Unit (ASLU) as a replacement for traditional linear layers to enhance modular expression. ASLU maintains the efficiency of linear transformations while incorporating learnable spline paths with local kernel response capabilities, thereby enhancing the structural expressiveness and improving input adaptability.

The input to the ASLU is a feature vector dimension $C_{\text{in}}$ , and the output is denoted as y . The overall output is composed of the sum of the outputs from the base linear path and the kernel-enhanced path. For relevant details, see (Equations 12–14):


$$y=y_{\mathrm{bs}}+y_{s\;\ln}$$ (12)

The base path $y_{\mathrm{bs}}$ follows the same formulation as a standard linear layer, expressed as:


$$y_{\mathrm{bs}}=W_{\text{base}}•\sigma (Z)$$ (13)

The weight matrix $W_{\text{base}}$ is a learnable linear parameter matrix, and σ(·) represents an element-wise activation function, typically utilizing continuous and differentiable functions such as SiLU or ReLU. This path captures the global linear structural relationships between inputs, ensuring computational efficiency in the base operation.

To enhance the structural expressiveness, ASLU introduces a kernel response path, which models regions of the input space that exhibit local structural variations or nonlinear response characteristics. This path defines a set of M B-spline kernel functions ${\left\{b_{t}(z_{j})\right\}}_{t=1}^{M}$ for each input dimension $z_{j}$ , forming a local support kernel basis. These kernel functions are then combined using a learnable kernel coefficient tensor, yielding the following output:


$$y_{\sin}=\sum_{j=1}^{C_{\text{in}}}\sum_{t=1}^{M}a_{j,t}\cdot b_{t}(z_{j})$$ (14)

Here, $a_{j,t}$ represents the output weight of the *t*-th spline basis function corresponding to the j-th input dimension, while M controls the number of kernel functions and their smoothing degree. Each B-spline kernel function has a non-zero response over a subinterval of its domain, thereby introducing sensitivity to local perturbations in the input. This enables the model to capture fine-grained structural details.

The two paths of the ASLU are ultimately combined in the output space through a channel-wise sum, allowing the model to leverage the global compositional advantage of the linear layer while also integrating the local expressiveness of the kernel path. The computational structure is highly parallelizable, ensuring excellent scalability and training stability. Furthermore, since the spline path is based on explicitly learnable kernel basis functions, the entire module exhibits strong interpretability, supporting visual analysis of the model’s response patterns across different regions of the feature space.

#### 2.2.3 Structure-aware multi-stage evolutionary optimization (SMEO)

In the task of crack image segmentation, model training often faces challenges such as uneven distribution of structural features, blurred boundary information, and unstable gradient responses, which in turn affect segmentation accuracy and training efficiency. To address these issues, we propose a Structure-aware Multi-stage Evolutionary Optimization (SMEO) strategy. The pseudocode for the SMEO optimization algorithm is presented in Algorithm 1. SMEO aims to guide the adaptive adjustment of model parameters through a three-stage dynamic feedback process in the feature space, including structure-aware exploration, feature-constrained fine-tuning, and global perturbation correction. This process enables a more efficient and structurally adaptive optimization procedure, thereby enhancing the model’s responsiveness to crack structure modeling.

**ALGORITHM 1:**
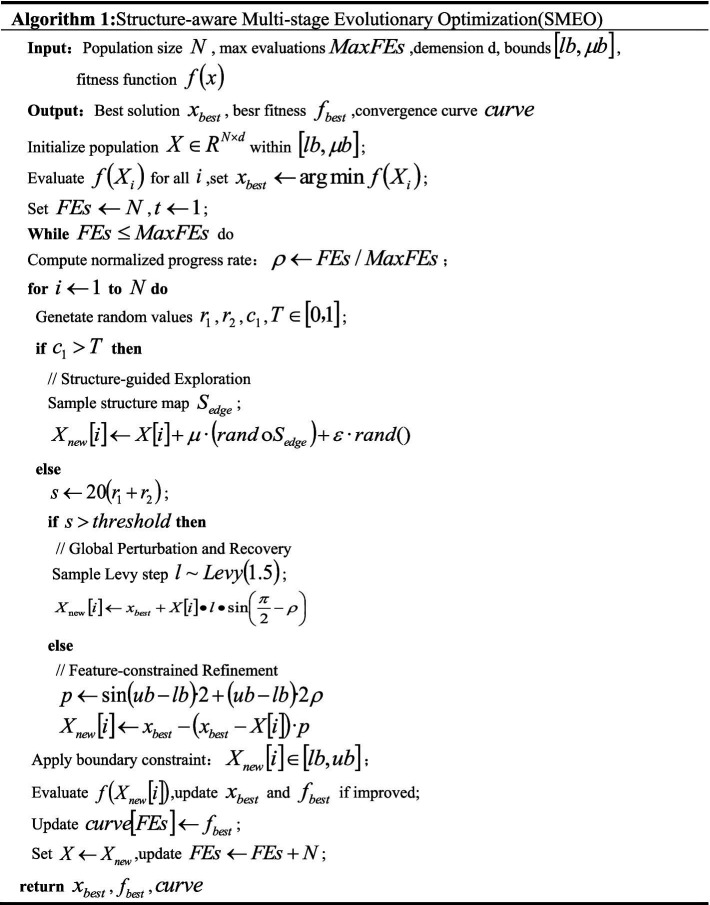
Schematic diagram of the SMEO optimization algorithm.

##### 2.2.3.1 Stage one: Structure-guided exploration

In the early stages of training, or when the feature distribution is still unstable, SMEO first activates the structure-aware exploration mechanism, guiding the optimization individuals to perform extensive sampling around the potential edge regions in the image. The first stage emphasizes the initial coverage of the target structural areas, preventing the model from getting trapped in local optima due to insufficient early gradients.

The update of the individual parameters is defined by the following formula. For relevant details, see (Equations 15–17):


$$x_{i}^{t+1}=x_{i}^{t}+\mu \cdot (r_{i}\cdot S_{\mathrm{eg}})+\in \cdot u$$ (15)

Here, $r_{i}$ represents the directional disturbance vector, $S_{\mathrm{eg}}$ is the structural edge response map derived from the current feature map, μ controls the strength of the structural guidance, and u~U(−1,1) denotes the uniform disturbance term. The first stage integrates spatial structural cues with global disturbance capabilities, thereby enhancing the model’s exploratory breadth and structural sensitivity in the early stages of training.

##### 2.2.3.2 Stage two: Feature-constrained refinement

As training progresses and the model’s perception of crack textures and boundaries stabilizes, SMEO transitions to the local refinement stage, guided by the optimal solution. During the feature-constrained fine-tuning phase, the optimization process focuses on modeling feature consistency and edge continuity. This is achieved by steering the individuals closer to the current global optimum, allowing for a more precise approximation of high-response regions. The update mechanism is as follows:


$$x_{i}^{t+1}=x_{g}^{t}-\eta \cdot [w_{t}\cdot (x_{g}^{t}-x_{i}^{t})+\delta \cdot \nabla_{s}]$$ (16)

Here, $x_{g}^{t}$ represents the current optimal individual, $w_{t}=\frac{t}{T_{\max}}$ is the self-adaptive convergence factor, and $\nabla_{s}$ denotes the structural gradient information derived from the feature map. The feature-constrained fine-tuning update strategy facilitates a smooth transition from coarse-grained perception to fine segmentation by coupling the structural guidance term with the positional discrepancy, thereby further enhancing the segmentation accuracy of boundary regions.

##### 2.2.3.3 Stage three: global perturbation and recovery

When the training process encounters performance bottlenecks or the model reaches a local convergence state, SMEO initiates the global disturbance mechanism. This mechanism enhances the diversity of the parameter search through an asymmetric jump strategy, enabling the model to escape local optimum regions. To minimize excessive disruption to training stability, the disturbance amplitude is controlled using a logarithmic suppression model. The disturbance update strategy is defined as follows:


$$x_{i}^{t+1}=x_{g}^{t}+k\cdot \mathrm{sign}(n)\cdot \log(1+|n|)$$ (17)

Here, k represents the disturbance amplitude coefficient, and $n\sim N(0,\sigma^{2})$ denotes a zero-mean Gaussian distribution variable. The global disturbance correction strategy effectively enhances the model’s adaptability to non-stationary regions, such as fracture patterns and blurred boundaries, during the later stages of training, thereby improving its overall ability to recognize structural integrity.

In summary, SMEO dynamically adapts the optimization behavior to the feature response states, thereby overcoming the drawbacks of the “single search mode throughout the entire process” commonly found in traditional optimization algorithms. Through stage-wise strategy transitions driven by structural perception, SMEO automatically switches to the most suitable optimization behavior based on the model’s training state, thereby enhancing training efficiency and improving the model’s ability to fit complex fracture structures.

## 3 Results

### 3.1 Experimental setup

To ensure the reliability of the RSA-TransUNet experimental results and eliminate potential confounding factors arising from environmental differences, all experiments in this study were conducted on the same hardware and software platform. In terms of software, a unified development environment was utilized, including a specific version of the operating system and a set of software tools, to maintain experimental consistency. To simplify the network output, BCEWithLogitsLoss was selected to handle the problem data caused by imbalance. (BCEWithLogitsLoss combines the Sigmoid activation function and binary cross-entropy loss). It avoids the problems of large loss function values or vanishing gradients at probability extremes (0 or 1). The number of training rounds is 300. A detailed list of the hardware configuration and software settings used for the experiments is provided in Table 1.

**Table 1:** Hardware and software parameters.

Hardware environment	CPU	Intel(R) Core(TM) i9-10980XE CPU @ 3.00GHz 3.00 GHz
	GPU	NVIDIA GeForce RTX 2080 Ti
	RAM	32.0 GB
	Video Memory	32GB
Software environment	OS	Windows 10
	CUDA Toolkit	V10.2
	CUDNN	V8.2.0
	Pytorch	1.8.1

### 3.2 Evaluation indicators

To comprehensively evaluate the model’s performance, four evaluation metrics were employed: Dice, IoU, Accuracy, and Recall, ensuring the accuracy of the experiments. First, four region definitions were introduced: True Positives (TP) – areas where cracks are present and predicted as such; True Negatives (TN) – areas where no cracks are present and predicted as such; False Positives (FP) – areas where no cracks are present but predicted as having cracks; and False Negatives (FN) – areas where cracks are present but predicted as not having cracks, as detailed in Table 2 (Zheng et al., 2025).

**Table 2:** Four regional definitions for segmentation results.

Labeled	Predicted	Regional definitions
Positive	Positive	TP
Positive	Negative	FN
Negative	Positive	FP
Negative	Negative	TN

Recall measures the proportion of actual positive samples that are correctly predicted. The calculation formula for Recall is as Equation 18. There are two possible outcomes: one is when the true positive (TP) class is correctly predicted, and the other is when the true positive (FN) class is predicted as negative. This metric represents the ratio of all tomato leaf images that are considered for segmentation relative to all those that truly require segmentation.


$$\text{Recall}=\frac{\mathrm{TP}}{\mathrm{TP}+\mathrm{FN}}$$ (18)

Precision refers to the ratio of the number of samples correctly predicted as positive to the total number of samples predicted as positive by the model. The calculation formula for Precision is as Equation 19:


$$\text{Precision}=\frac{\mathrm{TP}}{\mathrm{TP}+\mathrm{FP}}$$ (19)

The advantage of the F1-score lies in its ability to combine both Precision and Recall, providing a balanced evaluation of model performance. Since improving precision may lead to a reduction in recall, the F1-score is particularly useful for assessing model performance in imbalanced classification problems. The calculation formula for F1-score is as Equation 20.


$$F1-\text{score}=\frac{2{\text{Precision}}^{*}\text{Recall}}{\text{Precision}\;+\text{Recall}}$$ (20)

IoU refers to the ratio of the intersection of the actual and predicted areas. In this context, it represents the proportion of the overlap between the tomato disease regions and the corresponding labeled areas.


$$\mathrm{IoU}=\frac{\mathrm{TP}}{\mathrm{TP}+\mathrm{FN}+\mathrm{FP}}$$ (21)

Accuracy represents the proportion of correctly segmented tomato leaf images relative to the total number of correctly and incorrectly segmented samples in the dataset.


$$\text{Accuracy}=\frac{\mathrm{TP}+\mathrm{TN}}{\mathrm{TP}+\mathrm{FP}+\mathrm{TN}+\mathrm{FN}}$$ (22)

### 3.3 Experiment and discussion

#### 3.3.1 Ablation experiment

To ensure the fairness of the RSA-TransUNet experimental results and the stability of the network, ablation experiments involving three methods were conducted on the Crack500 crack dataset. This experiment utilized a controlled variable approach, combining ASMA, ASLUand SMEO for seven sets of ablation experiments. The F1-score was used as the primary evaluation metric, and the experimental results are recorded in Tables 3–5.

**Table 3:** Effectiveness of RSA-TransUNet.

Method	ASMA	ASLU	SMEO	Recall/%	F1-score/%	IoU/%	Accuracy/%
1	-	-	-	89.72	89.20	84.45	94.35
2	√	-	-	90.56	89.62	84.66	94.42
3	-	√	-	91.98	89.78	84.89	94.51
4	-	-	√	91.45	89.89	84.97	94.45
5	√	√	-	91.30	89.95	85.21	95.77
6	√		√	91.60	89.98	85.56	95.20
7	√	√	√	**91.62**	**90.10**	**85.54**	**95.80**

Bold indicates that our experimental parameters have reached the best level, playing a prominent role.

**Table 4:** Effectiveness of RSA-TransUNet (DeepCrack).

Method	ASMA	ASLU	SMEO	Recall/%	F1-score/%	IoU/%	Accuracy/%
1	-	-	-	79.02	77.99	74.15	84.23
2	√	-	-	79.76	78.29	74.32	84.34
3	-	√	-	80.41	79.13	74.56	84.56
4	-	-	√	80.98	79.34	75.14	85.72
5	√	√	-	81.45	80.52	75.46	85.89
6	√		√	82.13	81.12	75.97	86.15
7	√	√	√	**82.51**	**81.45**	**76.89**	**86.24**

Bold indicates that our experimental parameters have reached the best level, playing a prominent role.

**Table 5:** Effectiveness of RSA-TransUNet (CFD).

Method	ASMA	ASLU	SMEO	Recall/%	F1-score/%	IoU/%	Accuracy/%
1	-	-	-	87.01	86.01	78.21	87.13
2	√	-	-	87.56	86.25	78.54	87.62
3	-	√	-	88.23	87.02	78.69	88.14
4	-	-	√	88.47	87.13	78.97	88.79
5	√	√	-	89.41	87.95	79.13	89.52
6	√		√	89.52	88.21	80.02	89.98
7	√	√	√	**89.62**	**88.56**	**80.65**	**90.09**

Bold indicates that our experimental parameters have reached the best level, playing a prominent role.

In our ablation study comparisons, the ASMA module achieved a 0.42% improvement in image data performance. This enhancement can be attributed to the incorporation of directional awareness through the multi-path axial perturbation operation within ASMA, which, when combined with the Criss-Cross spatial attention mechanism, effectively captures long-range dependencies within the crack row-column structure. Furthermore, the incorporation of the ASLU network resulted in a 0.58% improvement over the baseline model. This enhancement is attributed to the efficiency and linear combination capabilities maintained by the fundamental linear paths in ASLU, while the local kernel response mechanism, constructed using B-spline basis functions, significantly enhances the model’s ability to capture and learn multi-class features of road cracks. Finally, the application of SMEO resulted in a 0.69% improvement, demonstrating that the model’s ability to learn road crack segmentation features has been effectively optimized across the three stages. Upon integrating the entire network into the model, the F1-score increased by 0.9%. These experimental results provide a clear indication of the exceptional segmentation performance of the RSA-TransUNet model. In the study of the Deepcrack dataset (see Table 4), after adding ASMA, the Recall metric of the model increased by 0.74%, the F1 metric increased by 0.3%, the IoU increased by 0.17%, and the Accuracy increased by 0.11%. This is due to the fine-grained construction of multi-scale crack texture features, making the model more sensitive to texture features. After adding ASLU, the Recall index of the model increased by 1.22%, the F1 index increased by 1.01%, the IoU increased by 0.48%, and the Accuracy increased by 1.01%. This is attributed to the high response representation of the linear variation module to weak crack variations. After adding SMEO, the Recall metric of the model increased by 1.46%, the F1 metric increased by 1.12%, the IoU increased by 0.76%, and the Accuracy increased by 1.49%. This indicates that the SMEO optimization algorithm can still improve during the process of enhancing the training speed. When the three modules act together, RSA-TransUNet has the greatest growth in the Recall metric, increasing by 3.49 to 82.51%, which is lower than 91.62% in the crack500 dataset. This might be because the resolution of the crack500 dataset is 2,560 × 2,592. Compared with the Deepcrack384 × 544 resolution image dataset, the model can better capture the transformation features of fine cracks, improve the feature extraction ability of the model, and is different from the Crack500 and CFD datasets. The Deepcrack dataset contains more multi-branch fine cracks and is more difficult to learn. In the study of ablation on CFD datasets (see Table 5), when the three modules acted together, the Recall metric of RSA-TransUNet reached 89.62%, the F1 metric reached 88.56%, the IoU metric reached 80.65%, and the Accuracy metric reached 90.09%. They are, respectively, slightly lower than 2, 1.54, 4.89 and 5.71% of the crack500 dataset. This might be because the small-scale dataset only contains 118 crack datasets, and the model fails to learn the complete and slightly varying crack features.

#### 3.3.2 Contrast experiment

To validate the effectiveness and advancement of the proposed algorithm, we selected classical segmentation networks and state-of-the-art models for comparison on the crack dataset, in order to demonstrate the generalization ability and superiority of the RSA-TransUNet network. To ensure a fair comparison, all models were trained for 180 epochs, as shown in Tables 6, 7.

**Table 6:** Compare the experimental results of different classification networks (crack500).

Models	Recall/%	F1-score/%	IoU/%	Accuracy/%
DeepLabV3 (Yurtkulu et al., 2019)	87.46	86.72	82.89	92.17
Segnet (Badrinarayanan et al., 2017)	87.65	87.98	83.46	93.26
PSPNet (Zhu et al., 2021)	88.36	88.46	84.32	94.13
TransUNet (Chen et al., 2021)	89.72	89.20	84.45	94.35
SwintNet (Cao et al., 2022)	90.56	89.13	84.65	94.67
Hybrid-Segmentor (Goo et al., 2025)	90.88	89.72	84.97	94.89
RSA-TransUNet	**91.62**	**90.10**	**85.54**	**95.80**

Bold indicates that our experimental parameters have reached the best level, playing a prominent role.

**Table 7:** Compare the experimental results of different classification networks (CFD).

Models	Recall/%	F1-score/%	IoU/%	Accuracy/%
DeepLabV3 (Yurtkulu et al., 2019)	85.76	85.70	80.65	90.09
Segnet (Badrinarayanan et al., 2017)	85.97	85.89	80.84	90.18
PSPNet (Zhu et al., 2021)	86.67	86.38	81.63	91.54
TransUNet (Chen et al., 2021)	87.00	86.59	82.22	92.19
SwintNet (Cao et al., 2022)	87.02	86.97	82.98	92.02
Hybrid-Segmentor (Goo et al., 2025)	88.65	88.41	82.66	93.15
RSA-TransUNet	**89.62**	**88.56**	**83.54**	**93.46**

Bold indicates that our experimental parameters have reached the best level, playing a prominent role.

As shown in Table 6, on the classic road crack Crack500 public dataset, RSA-TransUNet achieved the best performance. Compared to the second-best model, it improved Recall, F1-score, Precision, and Accuracy by 0.74, 0.38, 0.57, and 0.91%, respectively. These experimental results demonstrate the superiority of the model.

Table 7 presents a comparison between RSA-TransUNet and other segmentation networks on the classic road crack CFD public dataset. It can be seen that our proposed model achieved the best performance, with improvements of 0.97, 0.15, 0.88, and 0.31% in Recall, F1-score, Precision, and Accuracy, respectively, compared to the second-best model. In conclusion, the results from both experiments further validate the advancement and effectiveness of the model.

**Figure 3:**
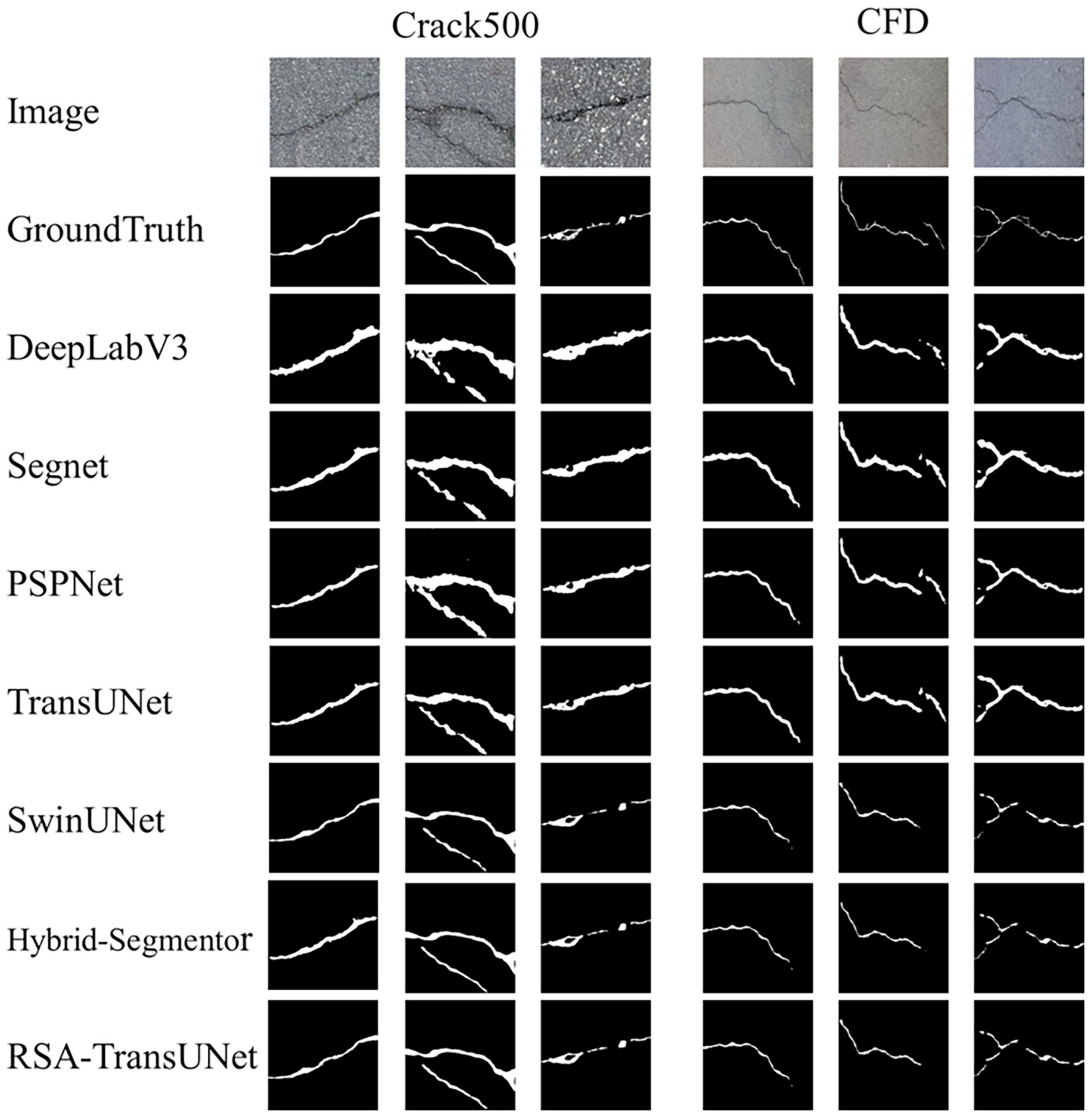
The segmentation results of different models on the Crack500 and CFD datasets.

As shown in Figure 3, we compared the segmentation performance of the DeepLabV3, Segnet, PSPNet, TransUNet, SwintNet, and Hybrid-Segmentor networks on the Crack500 and CFD datasets. A detailed analysis is as follows:

The segmentation performance of DeepLabV3 was the poorest, as it incorrectly segmented non-crack regions as crack regions in the third column, which may be attributed to the inclusion of Conditional Random Fields (CRF) within the model. While CRFs improve boundary precision, they do not fully address the detail-related issues in high-resolution images. The segmentation accuracies for DeepLabV3 on the Crack500 dataset were 87.46, 86.72, 82.89, and 92.17%. Furthermore, SegNet’s segmentation results showed little difference compared to DeepLabV3. However, in cases involving intersecting or overlapping cracks (such as in the third column of crack images), SegNet achieved segmentation accuracies of 87.65, 87.98, 83.46, and 93.26%. This may be due to its typical encoder-decoder architecture, which is more suitable for lower-resolution input images and struggles to capture global contextual information. In particular, when handling complex backgrounds and intricate details, SegNet tends to produce blurry segmentation results. PSPNet, leveraging its pyramid pooling module, aggregates image information across multiple scales, resulting in superior performance compared to the first two models. However, its segmentation accuracy remains relatively low on the CFD micro-crack dataset (e.g., the sixth column of crack images in Figure 3), with segmentation accuracies of 88.36, 88.46, 84.32, and 94.13%. The TransUNet model, serving as the baseline for this study, demonstrates strong global crack detection capabilities, achieving segmentation accuracies of 89.72, 89.20, 84.45, and 94.35%. Nonetheless, it struggles to capture subtle variations in crack features. SwinTNet, with its hierarchical Transformer design, effectively captures features at multiple scales from low to high layers. It exhibits greater flexibility when handling diverse crack images, yielding segmentation accuracies of 90.56, 89.13, 84.65, and 94.67%. Finally, the Hybrid-Segmentor, as an advanced segmentation network, demonstrates a significant improvement in crack segmentation performance compared to the previous models. This enhancement is likely due to the model’s ability to balance the capture of both local and global contextual information, especially in terms of detail processing and large-scale context understanding. The segmentation accuracies achieved are 90.88, 89.72, 84.97, and 94.89%. Our proposed RSA-TransUNet model, which excels in capturing fine details and textures, particularly on the CFD micro-crack dataset, achieves the best results (segmentation accuracies of 91.62, 90.10, 85.54, and 95.80%). In conclusion, the experimental results presented in Figure 3 demonstrate that traditional segmentation models exhibit similar performance when handling conventional, well-defined cracks. However, when applied to small, intertwined, or edge-blurred crack images, the proposed RSA-TransUNet model achieves superior accuracy, outperforming other state-of-the-art segmentation networks, particularly in terms of Recall, where a notable improvement is observed.

**Figure 4:**
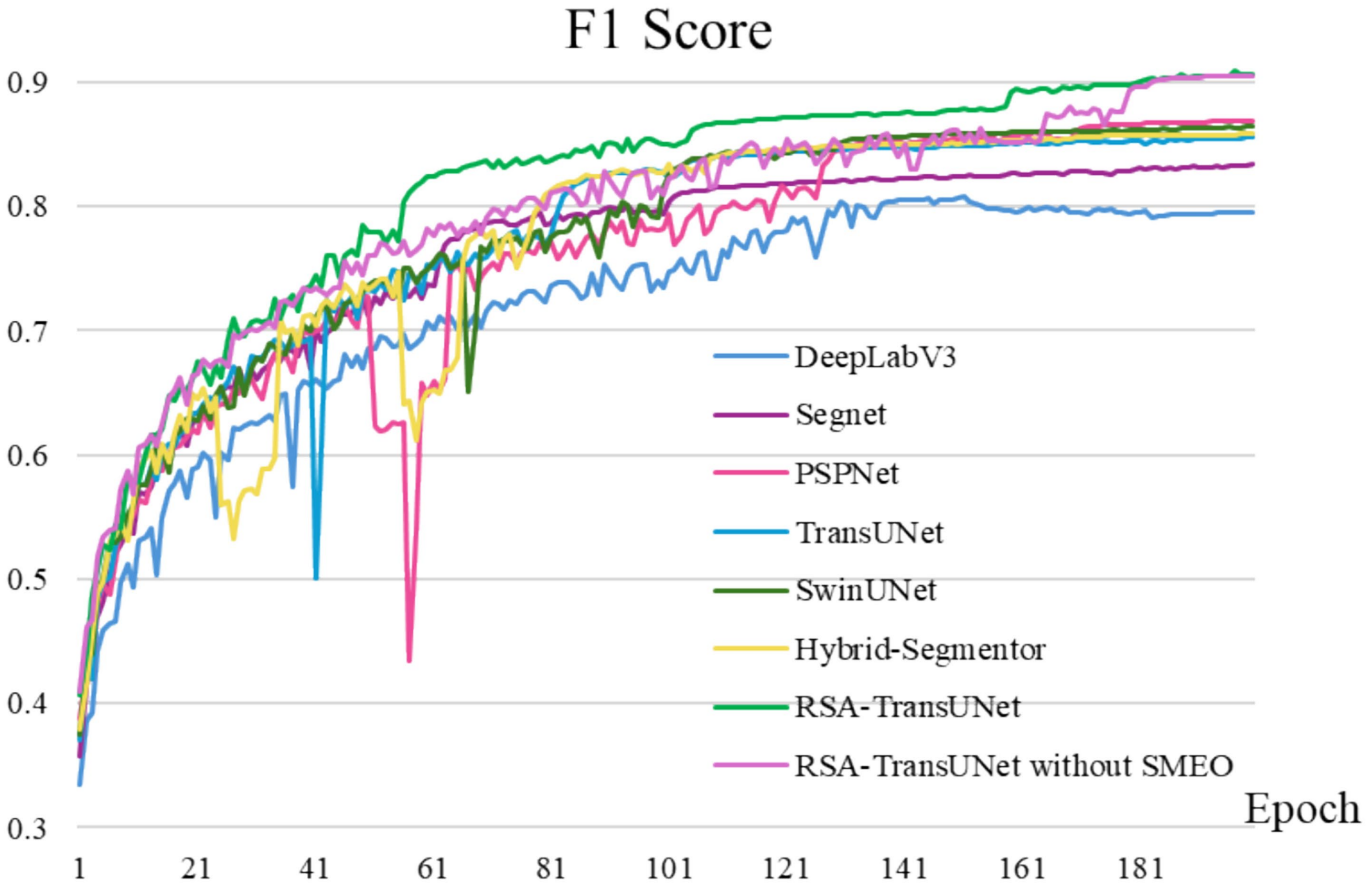
Comparative experiment of models.

As shown in Figure 4, we set the model training to 180 epochs. After incorporating SMEO, the model exhibits a convergence trend around the 60th epoch, which significantly accelerates the convergence speed compared to other segmentation models that converge around the 120th epoch. Moreover, at the 60th epoch, the F1-score of RSA-TransUNet reaches approximately 85.0%, which is substantially higher than the 77.5% achieved by other networks at the same stage. These experimental results effectively validate the efficacy of the proposed SMEO method, while also demonstrating that RSA-TransUNet addresses the challenge of high training costs associated with large-scale datasets, achieving a balance between accuracy and speed.

#### 3.3.3 Generalization experiment

**Figure 5:**
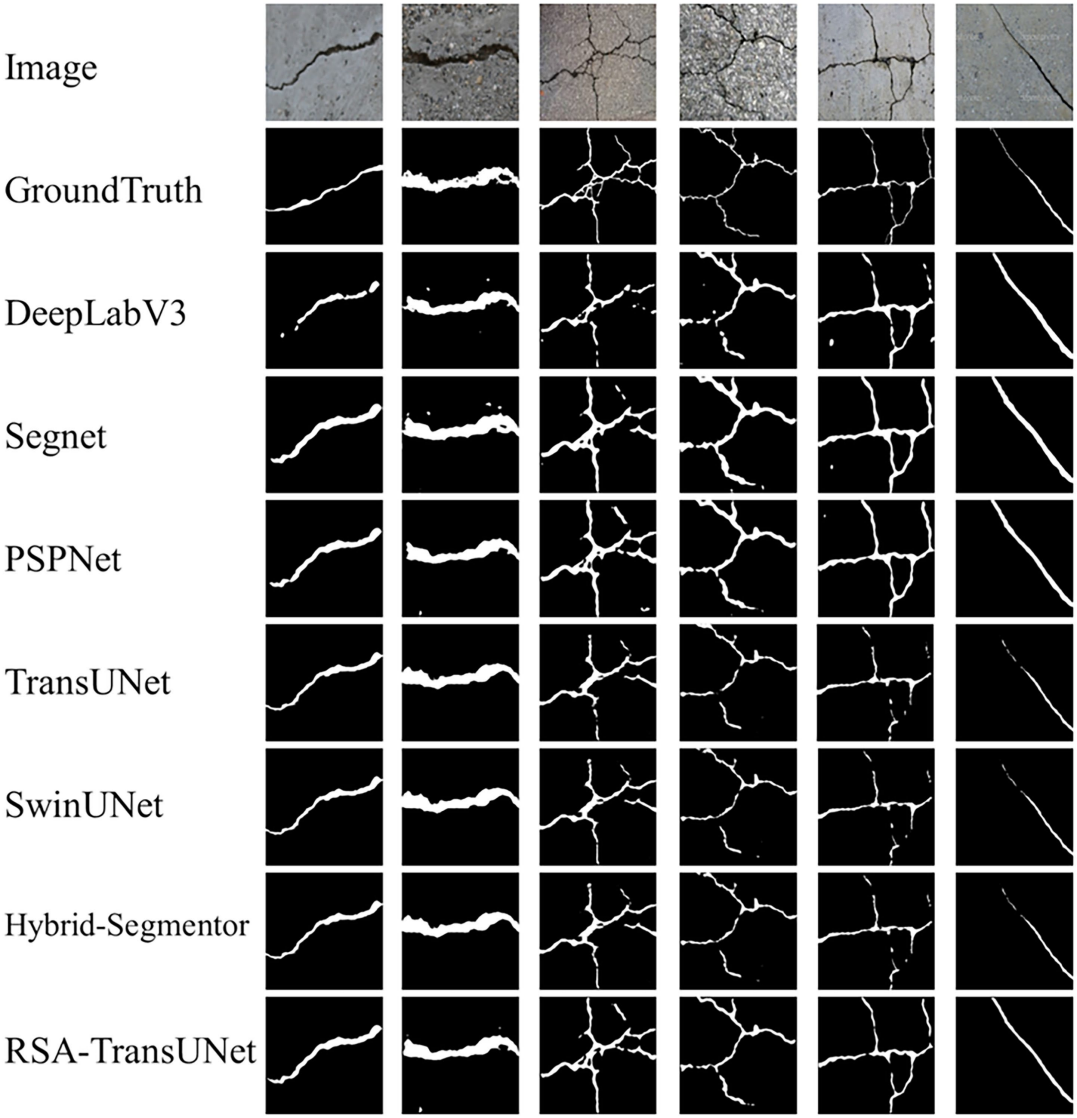
Generalization experiment segmentation map of RSA-TransUNet on the Deepcrack dataset.

To evaluate the generalization capability of RSA-TransUNet, we conducted generalization experiments on the Deepcrack public crack dataset. Figure 5 illustrates six types of crack features with distinct characteristics, including left-sloping upward cracks, horizontal-width cracks, mesh-interwoven cracks, I-shaped cracks, wide-to-narrow cracks, and right-sloping downward cracks. Classical segmentation models, such as DeepLabV3, SegNet, and PSPNet, demonstrate good performance when handling wide cracks or simple single-line cracks. In contrast, models like TransUNet, SwinUNet, Hybrid-Segmentor, and RSA-TransUNet show higher accuracy in segmenting multi-scattered, small, and fine cracks. These experimental results indicate that the RSA-TransUNet model outperforms other segmentation networks in terms of stability and precision, with an F1-score improvement of 1.2% over the second-best model.

**Figure 6:**
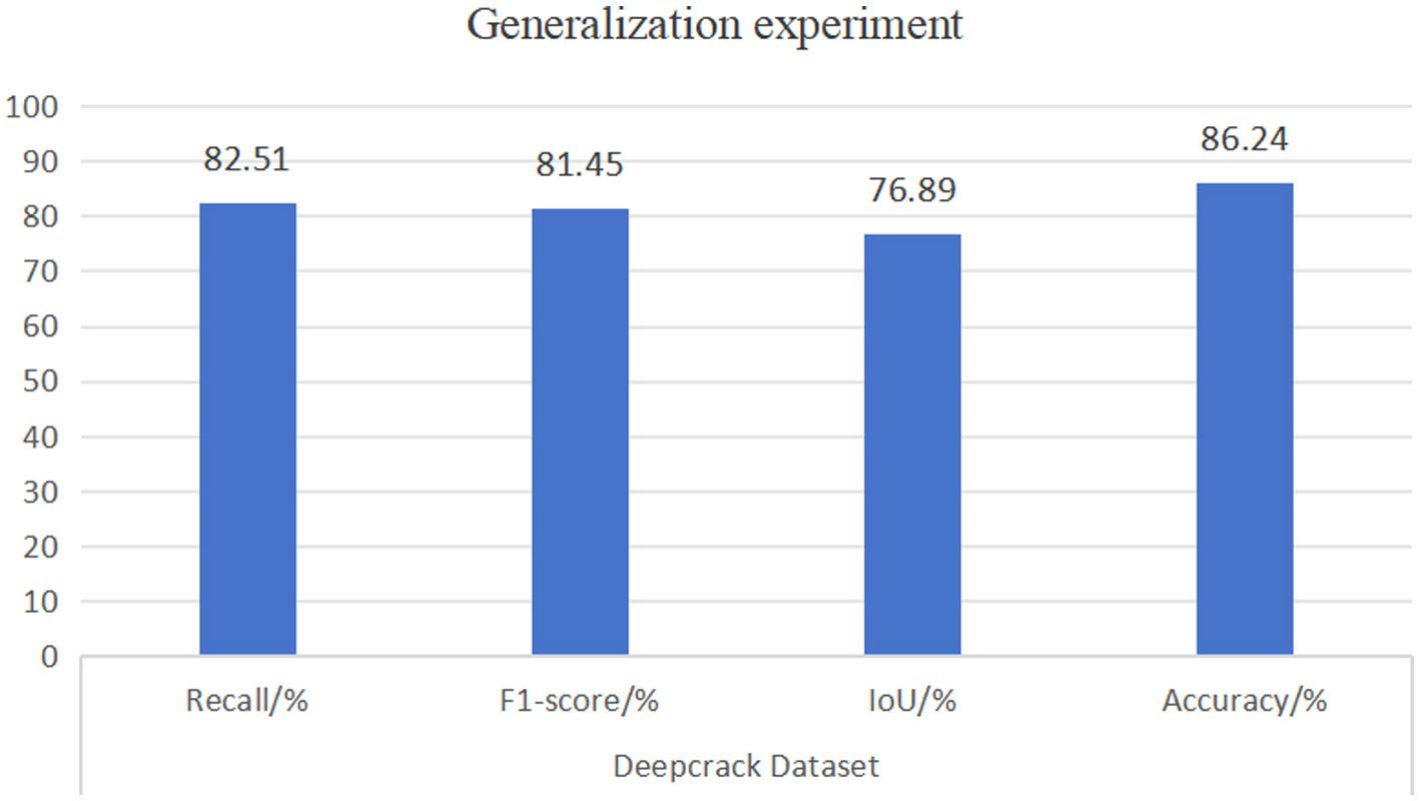
Result graph of the generalization experimental dataset.

Table 8 shows the comparison results of the Deepcrack dataset with other models in RSA-TransUNet. It can be seen that the Recall, F1, IoU, and Accuracy have reached 82.51, 81.45, 76.89, and 86.24% respectively, which are 2.3% higher than the second best model, respectively. 1.2, 0.86, 0.24%. The experimental results further verify that the RSA-TransUNet we proposed is advanced and has high segmentation efficiency (Figure 6).

**Table 8:** Compare the experimental results of different classification networks (Deepcrack).

Models	Recall/%	F1-score/%	IoU/%	Accuracy/%
DeepLabV3 (Yurtkulu et al., 2019)	77.19	78.43	71.97	80.43
Segnet (Badrinarayanan et al., 2017)	77.67	78.96	72.45	81.58
PSPNet (Zhu et al., 2021)	78.29	79.38	73.67	82.12
TransUNet (Chen et al., 2021)	79.24	79.15	74.82	83.46
SwintNet (Cao et al., 2022)	79.38	80.22	75.41	84.15
Hybrid-Segmentor (Goo et al., 2025)	80.21	80.25	76.03	86.00
RSA-TransUNet	**82.51**	**81.45**	**76.89**	**86.24**

Bold indicates that our experimental parameters have reached the best level, playing a prominent role.

## 4 Conclusion

This paper proposes a novel network for complex road crack segmentation, named RSA-TransUNet. RSA-TransUNet is built on the nested structure of Transformer and Unet, combining the strengths of both architectures. To address challenges such as edge-blurred crack texture features, multi-class crack segmentation, and slow training speed on large-scale crack datasets, the paper introduces ASMA, ASLU, and SMEO. The results of ablation, comparative, and generalization experiments demonstrate that RSA-TransUNet outperforms six classical and state-of-the-art segmentation networks. Specifically, the model achieves a Recall of 91.62%, an F1-score of 90.10%, a Precision of 85.54%, and an Accuracy of 95.80%. The proposed method holds significant promise for applications in engineering, with the potential for further adaptation to various use cases. Additionally, the model is highly effective for crack detection in other types of infrastructure.

However, it is important to note that while the proposed RSA-TransUNet effectively addresses the segmentation of fine textures in edge regions, several issues remain: (1) The model has a relatively large number of parameters, which, despite enabling rapid optimization during training, poses challenges for hardware deployment due to the high computational demand; (2) The segmentation accuracy for elongated, interwoven cracks with extremely fine textures is lower, although the overall segmentation performance still outperforms existing models. Future work will focus on the following areas: (1) Reducing the model size and the number of parameters to achieve faster segmentation and improved accuracy; (2) Expanding the dataset of fine, interwoven crack textures to enhance the model’s learning capability.

In conclusion, the RSA-TransUNet segmentation model demonstrates high efficiency and accuracy in detecting road crack defects, with the segmented regions closely aligning with actual crack areas. Looking ahead, we aim to integrate Internet of Things (IoT) sensor technology with image segmentation techniques to develop a more refined crack segmentation approach, facilitating the creation of a precise and efficient crack detection system. The introduction of RSA-TransUNet provides a valuable reference for the future development of intelligent transportation technologies and offers a novel, intelligent solution to address road safety incidents caused by crack defects.

## Data Availability

The original contributions presented in the study are included in the article/supplementary material, further inquiries can be directed to the corresponding author.
