# Correction: RSA-TransUNet: a robust structure-adaptive TransUNet for enhanced road crack segmentation

**DOI:** 10.3389/fnbot.2025.1711642

**Published:** 2025-11-10

**Authors:** Liling Hou, Fei Yu, Yaowen Hu, Yang Hu, Ruoli Yang

**Affiliations:** 1 Zhangzhou Institute of Technology, Zhangzhou, China; 2 College of Physics and Information Engineering, Minnan Normal University, Zhangzhou, China; 3 Key Lab of Light Field Manipulation and System Integration Applications in Fujian Province, School of Physics and Information Engineering, Minnan Normal University, Zhangzhou, China; 4 Key Lab of Intelligent Optimization and Information Processing, Minnan Normal University, Zhangzhou, China; 5 College of Computer Science and Technology, National University of Defense Technology, Changsha, China; 6 College of Electronic Information and Physics, Central South University of Forestry and Technology, Changsha, China; 7 College of Systems Engineering, National University of Defense Technology, Changsha, China

**Keywords:** road crack segmentation, axial-shift MLP attention, adaptive spline linear unit, structure-aware multi-stage, evolutionary optimization

In the published article, in the Funding section, the funding sources “Education Research Project, JAT210843 and Zhangzhou Institute of Technology Project, ZZY2021b039 to Liling Hou” were erroneously omitted. The corrected Funding statement appears below:

The original version of this article has been updated.

